# Targeted therapy for Epstein-Barr virus-associated gastric carcinoma using low-dose gemcitabine-induced lytic activation

**DOI:** 10.18632/oncotarget.5041

**Published:** 2015-09-04

**Authors:** Hyun Gyu Lee, Hyemi Kim, Eun Jung Kim, Pil-Gu Park, Seung Myung Dong, Tae Hyun Choi, Hyunki Kim, Curtis R. Chong, Jun O. Liu, Jianmeng Chen, Richard F. Ambinder, S. Diane Hayward, Jeon Han Park, Jae Myun Lee

**Affiliations:** ^1^ Department of Microbiology and Immunology, Yonsei University College of Medicine, Seoul, Republic of Korea; ^2^ Brain Korea 21 PLUS Project for Medical Sciences, Yonsei University College of Medicine, Seoul, Republic of Korea; ^3^ Radiopharmaceutical Research Team, Korea Institute of Radiological and Medical Sciences, Seoul, Republic of Korea; ^4^ Research Institute, National Cancer Center, Goyang, Gyeonggi-do, Republic of Korea; ^5^ Department of Pathology, Yonsei University College of Medicine, Seoul, Republic of Korea; ^6^ Lowe Center for Thoracic Oncology, Dana-Farber Cancer Institute, Boston, Massachusetts, MA, USA; ^7^ Department of Medicine, Brigham and Women's Hospital, Boston, Massachusetts, MA, USA; ^8^ Department of Pharmacology and Molecular Sciences, Johns Hopkins University School of Medicine, Baltimore, MD, USA; ^9^ Department of Oncology, Johns Hopkins University School of Medicine, Baltimore, MD, USA

**Keywords:** Epstein-Barr virus-associated gastric carcinoma, gemcitabine, ataxia telangiectasia-mutated, p53, EBVaGC mouse model

## Abstract

The constant presence of the viral genome in Epstein-Barr virus (EBV)-associated gastric cancers (EBVaGCs) suggests the applicability of novel EBV-targeted therapies. The antiviral nucleoside drug, ganciclovir (GCV), is effective only in the context of the viral lytic cycle in the presence of EBV-encoded thymidine kinase (TK)/protein kinase (PK) expression. In this study, screening of the Johns Hopkins Drug Library identified gemcitabine as a candidate for combination treatment with GCV. Pharmacological induction of EBV-TK or PK in EBVaGC-originated tumor cells were used to study combination treatment with GCV *in vitro* and *in vivo*. Gemcitabine was found to be a lytic inducer via activation of the ataxia telangiectasia-mutated (ATM)/p53 genotoxic stress pathway in EBVaGC. Using an EBVaGC mouse model and a [^125^I] fialuridine (FIAU)-based lytic activation imaging system, we evaluated gemcitabine-induced lytic activation in an *in vivo* system and confirmed the efficacy of gemcitabine-GCV combination treatment. This viral enzyme-targeted anti-tumor strategy may provide a new therapeutic approach for EBVaGCs.

## INTRODUCTION

Epstein-Barr virus (EBV) is a double-stranded DNA human gamma herpes virus that establishes a persistent infection in over 90% of individuals. Most infections are self-limiting, but some cases are associated with the development of malignancies of lymphoid or epithelial origin [1]. EBV-associated gastric carcinomas (EBVaGCs) make up about 9% of all stomach cancers [2]. The presence of EBV in lymphoma or leukemia is known to confer a poorer prognosis [3–5]; however, a recent retrospective study revealed that EBV positivity in gastric cancer is associated with lower mortality and provides an additional prognostic indicator [2]. In addition to the conventional chemotherapy and surgical treatments, many EBV-positive malignancy experimental treatments are aimed at targeting the EBV episome, inhibiting EBV-transforming proteins, EBV-dependent expression of cellular toxins, and modulation of immune responses with EBV-specific cytotoxic T lymphocytes (CTL) [6, 7]. Recent studies based on the concept of selective destruction of tumor cells have suggested that the induction of lytic activation in EBV-associated tumors and combination treatment with the antiviral agent ganciclovir (GCV) represents a potential anti-cancer treatment modality [7–10].

GCV is efficiently phosphorylated and activated by the viral thymidine kinase (TK) or protein kinase (PK) [11, 12]. Phosphorylated GCV interferes with subsequent cellular DNA synthesis, resulting in apoptotic cell death [13, 14]. Herpes Simplex Virus (HSV)-TK/GCV cytotoxic gene therapy is effective *in vitro*, but the gene delivery of HSV-TK is problematic *in vivo* [15]. Endogenous EBV-TK or EBV-PK (referred to as EBV-TK/PK) induced during lytic activation in EBV-associated tumors, however, may provide an alternative strategy [16]. Therefore, identification of the reagents that can induce lytic activation in EBV-associated tumors is critical.

Several pharmacological agents are known to induce lytic activation via the endoplasmic reticulum (ER) or genotoxic stress response in EBV-infected cells [8, 9, 17–19]. We screened the Johns Hopkins Drug Library (JHDL) to find clinically applicable new drugs as a drug repositioning approach [20]. From this screen, we selected gemcitabine (2, 2-difluorodeoxycytidine, dFdC; Gemzar), which has been used in various cancer therapeutic regimens [21–24]. Gemcitabine has been shown to be a lytic inducer with therapeutic potential in EBV-positive B cell lymphoma cell lines and nasopharyngeal carcinoma cell lines [8, 25], but this drug has not been examined with respect to the precise mechanism of lytic activation in the context of EBVaGC.

In this study, we determined the dose of gemcitabine required for the induction of EBV lytic activation and explored the mechanism of this drug. Moreover, we determined whether gemcitabine-GCV combination treatment was effective in inducing cell death in SNU-719 cells, a gastric cancer cell line that is naturally infected with EBV. We established an EBVaGC-bearing mouse model and [^125^I]-1-(2-fluoro-2-deoxy-D-arabinofuranosyl)-5-iodouracil (FIAU)-based molecular imaging to evaluate gemcitabine-induced lytic activation and gemcitabine-GCV combination treatment *in vivo*. The effectiveness of combination therapy was confirmed *in vivo* by this mouse model and imaging system.

## RESULTS

### The expression of EBV-TK/PK during gemcitabine-induced lytic activation in SNU-719 cells

**Figure 1:**
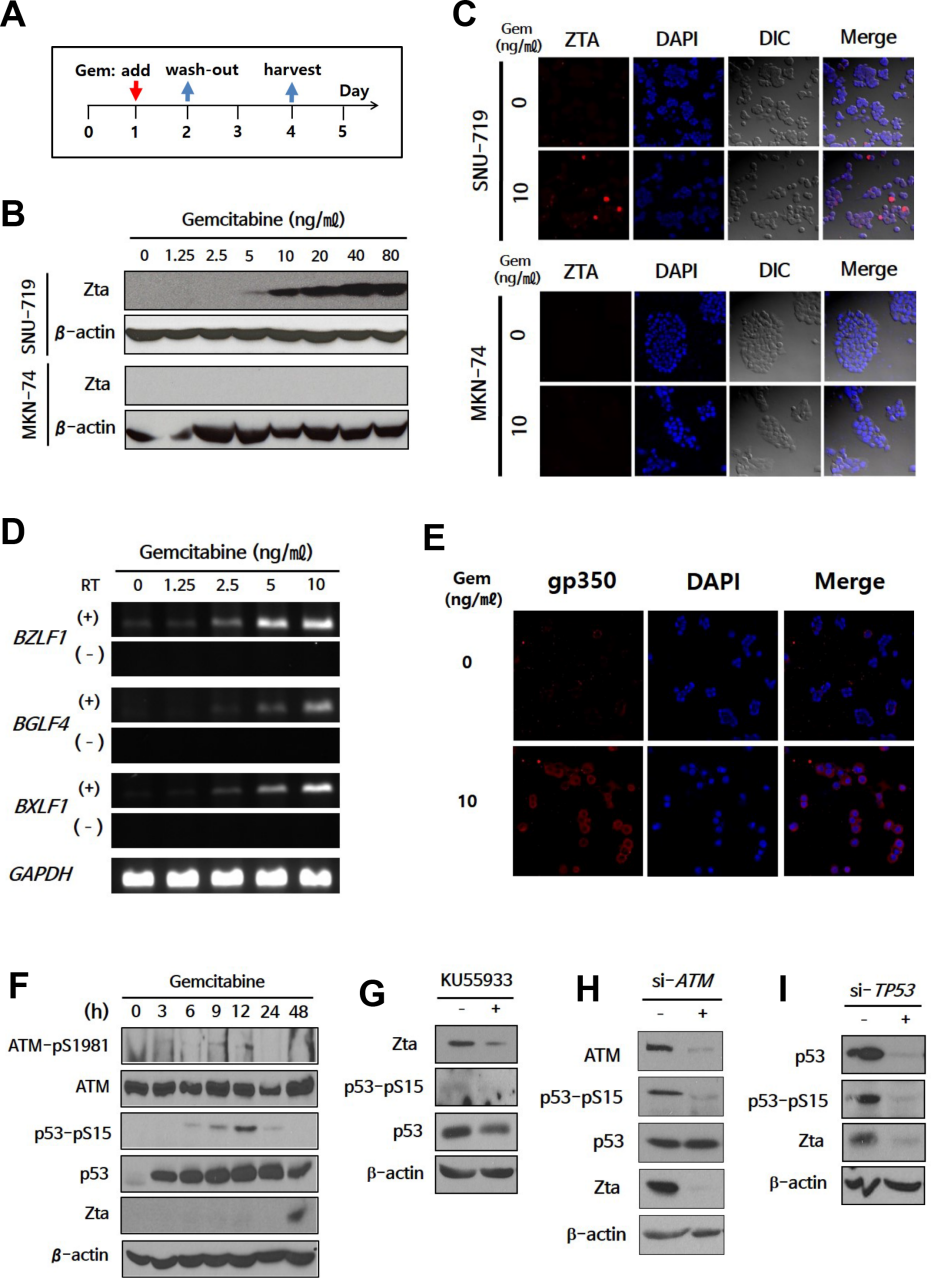
Expression of EBV-TK/PK during gemcitabine-induced lytic activation via ATM/p53 genotoxic stress pathway in EBVaGC cells **A.** Administration schedule of gemcitabine. EBVaGC cells (SNU-719) or EBVnGC cells (MKN-74) were treated with gemcitabine (0-80 ng/ml) for 24 h and were cultured for another 2 days. Zta expression was evaluated by western blot **B.** and IFA **C.** Blue, DAPI; Red, Zta. **D.** RT-PCR for *BZLF1*, *BGLF4*, and *BXLF1* was performed on gemcitabine (0–10 ng/ml)-treated SNU-719 cells. RT (−) lane is a negative control to determine contamination by EBV genomic DNA. **E.** gp350 was visualized by IFA in 10 ng/ml gemcitabine-treated SNU-719 cells. Blue, DAPI; Red, gp350. **F.** Changes in phosphorylated ATM (pSer 1981) and p53 (pSer 15) during gemcitabine treatment were evaluated by western blot. **G–I.** ATM inhibitor (KU55933) was treated after 24 h-gemcitabine treatment, while si-*ATM* and si-*TP53* were transfected before gemcitabine treatment. Inhibition of lytic activation by KU55933, si-*ATM*, or si-*TP53* was evaluated by changes in Zta, ATM, phosphorylated ATM, p53, and phosphorylated p53 using western blot. β-actin or *GAPDH* was used as loading controls.

We sought to identify new chemical reagents that could induce lytic activation in EBVaGCs by high-throughput screening of JHDL using EBV BZLF1 promoter-transfected human gastric carcinoma (AGS) cells [20]. From 2,687 drugs, we got 188 candidates showing significantly increased luciferase activity when compared with control (Supplementary Table S1). Validation experiments were performed on the upper 15% (29 drugs, bold lettering in Supplementary Table S1). Gemcitabine was identified as an ideal candidate for further evaluation. Treatment of the EBVaGC cell line SNU-719 and the EBV-negative gastric cancer (EBVnGC) cell line MKN-74 with gemcitabine as scheduled in Figure 1A revealed that the EBV immediate early (IE) lytic protein Zta was induced in SNU-719 cells even at a low dose (5 ng/ml; Figure 1B). Zta protein expression was confirmed by immunofluorescence microscopy (IFA) (Figure 1C). Moreover, this effect was observed beginning 48 h after gemcitabine treatment (Supplementary Figure S1A and S1B). To determine whether the low dose of gemcitabine induces other lytic genes, we performed RT-PCR to evaluate the induction of *BGLF4* (EBV-PK) and *BXLF1* (EBV-TK). These genes exhibited a similar expression pattern to that of *BZLF1*, which encodes the Zta (Figure 1D). Additionally, a component of virion, gp350, was detected only in lytic activation-induced SNU-719 cells (Figure 1E).

The ER or genotoxic stress response is associated with EBV lytic activation [18, 26]. Moreover, the ataxia telangiectasia-mutated (ATM) kinase/p53 pathway is activated during genotoxic stress-induced EBV lytic activation [18]. We first screened for the involvement of the ER stress response during gemcitabine-induced lytic activation, but C/EBP-homologous protein (CHOP) and glucose-regulated protein-78 (GRP78), which are known ER stress markers, exhibited no differences between SNU-719 and MKN-74 cells following gemcitabine treatment (Supplementary Figure S2). We next evaluated lytic activation in the context of ATM/p53 activation. SNU-719 cells have wild-type *TP53* [27], yielding an intact ATM/p53 pathway. Serine 1981 of ATM was phosphorylated 3 h after gemcitabine treatment, and serine 15 of p53 was phosphorylated subsequently (Figure 1F). Phosphorylated p53 was decreased following treatment with the ATM inhibitor KU55933 (Figure 1G), which may have suppressed Zta expression as previously reported [18]. To further evaluate the involvement of the ATM/p53 pathway in lytic activation, we performed siRNA-based knock-down experiments. Phosphorylation of p53 was decreased by si-*ATM*, resulting in a decrease of Zta protein expression (Figure 1H). Moreover, this finding was confirmed by si-*TP53* (Figure 1I). Collectively, these results suggest that gemcitabine induces lytic activation via the ATM/p53-mediated genotoxic stress pathway in SNU-719 cells.

### Gemcitabine confers GCV susceptibility on EBVaGC cells

**Figure 2:**
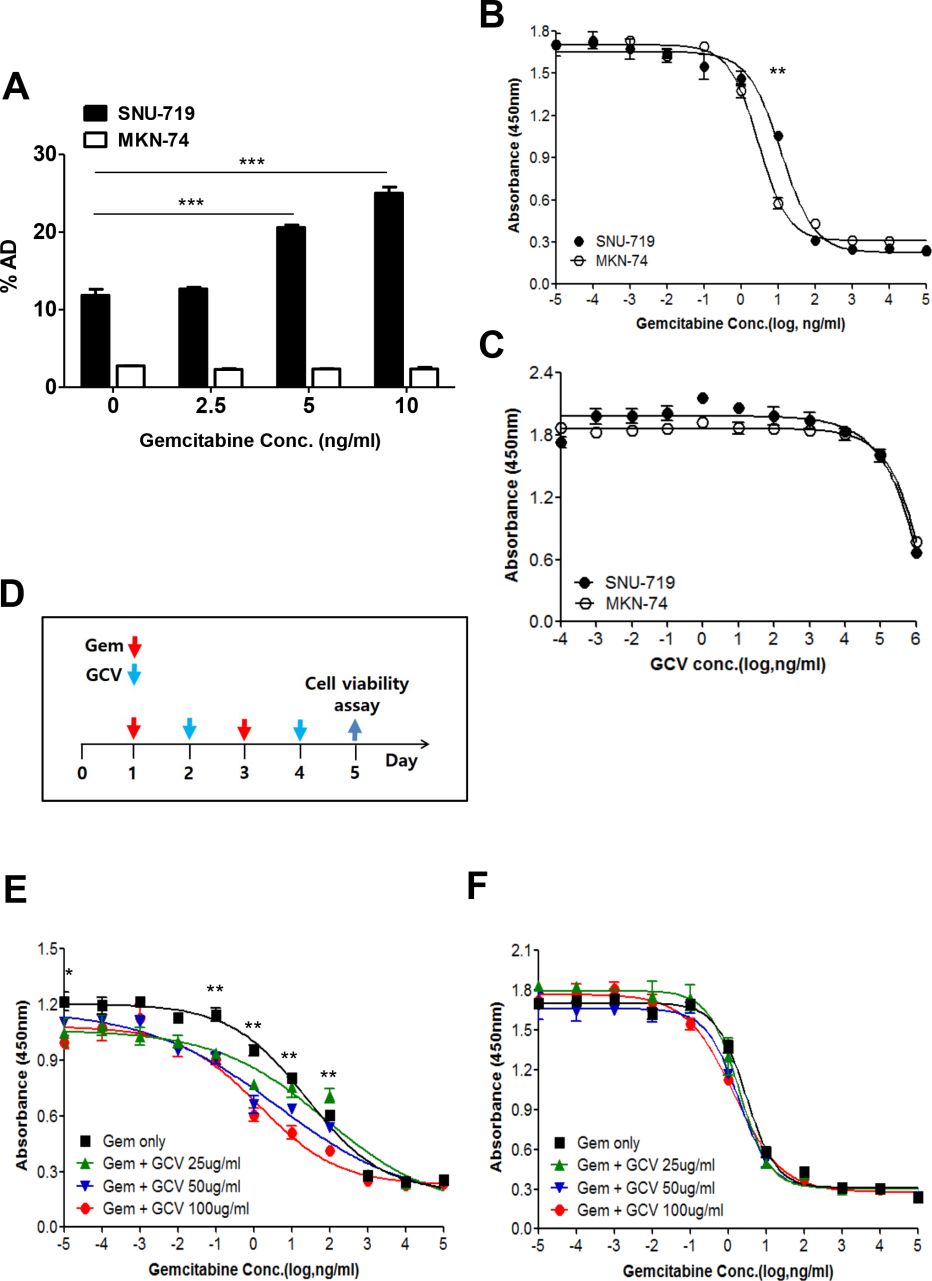
Gemcitabine confers GCV susceptibility on EBVaGC cells **A.** Gemcitabine-treated SNU-719 or MKN-74 cells were incubated with 1 μCi/2 ml [^125^I] FIAU for 4 h. The radioactivity of harvested cells was determined by a γ-counter. SNU-719 and MKN-74 cells were treated with either **B.** gemcitabine or **C.** GCV for 4 days. Then the viable cells were determined by the CCK-8 assay. **D.** Administration schedule of gemcitabine and GCV. Dose response of **E.** SNU-719 cells and **F.** MKN-74 cells to gemcitabine. Serially diluted gemcitabine and indicated doses of GCV were added as administration schedule. The 95% confidence intervals of the slopes, which were determined using best-fit four-parameter regression, are shown. The statistical analysis was performed between Gem only and Gem + 100 ug/ml GCV. Values represent means ± SEM. *P < 0.05, **P < 0.01, ^***^P < 0.001.

To confirm that the induction of EBV-TK/PK was applicable to this combination treatment, enzymatic activity was measured using the radio-isotope labeled-nucleoside analogue, [^125^I] FIAU [28]. Cellular accumulation of [^125^I] FIAU showed a positive correlation with the dose of gemcitabine in SNU-719 cells but not in MKN-74 cells (Figure 2A).

We examined the concentration of gemcitabine that induced lytic activation while minimizing cell death as gemcitabine is currently used as a chemotherapeutic drug in various kinds of cancers [21–24]. The inhibition of cell proliferation by 50% (IC_50_) for MKN-74 cells was 2.2–3.8 ng/ml, which is similar to the concentration previously reported [22], while that of SNU-719 cells (8.4–16.8 ng/ml) was slightly higher (Figure 2B). Thus, the induction of lytic activation in SNU-719 cells by 5 ng/ml gemcitabine occurred at a level below the IC_50_. To establish a combination treatment protocol with gemcitabine and GCV, we first treated cells with GCV alone. Little difference in the response of SNU-719 and MKN-74 cells to GCV treatment was noted, and GCV had little influence on both cell types even at a relatively high concentration (100 μg/ml; Figure 2C).

The cytotoxicity of the combination treatment was evaluated under an optimized schedule as described in Figure 2D. This schedule was based on the toxicity and short half-life (8–17 min) of gemcitabine [29]. Gemcitabine conferred cytotoxicity on GCV in SNU719 cells but not in MKN-74 cells (Figure 2E and 2F). GCV worked more efficiently in concert with the low concentration of gemcitabine. That is, the decrease in cell survival was more profound at 0.1–10 ng/ml than at 10–100 ng/ml. GCV did not exert an additional effect at gemcitabine concentrations of 1 μg/ml. GCV induced maximum efficacy when 100 μg/ml GCV was combined with lytic activation-inducible concentrations (1–100 ng/ml) of gemcitabine *in vitro*. Taken together, a low dose of gemcitabine combined with GCV is an efficacious combination treatment option in these cells.

### Establishment of a lytic activation-inducible SNU-719 cell-implanted NOD-SCID mouse model

An EBVaGC animal model system is indispensable for *in vivo* evaluation of gemcitabine-GCV combination treatment. We therefore developed a mouse model for this purpose using NOD-SCID. SNU-719 cell-implanted mice developed measurable tumors in 40–45 days after implantation. Although Matrigel did not affect cell viability, Matrigel-based SNU-719 tumors were more suitable for *in vivo* experiments than PBS-based inocula because of improved tumor establishment efficiency and consistency of tumor size (Supplementary Figure S3A). Isolated tumors exhibited necrotic regions as reported previously (Supplementary Figure S3B) [30, 31]. Moreover, tumors kept the phenotypes of SNU-719 cells and EBV genome, which was verified by flow cytometry (Supplementary Figure S3C) and EBV-encoded small RNAs (EBER)-*in situ* hybridization (ISH) (Supplementary Figure S3D).

**Figure 3:**
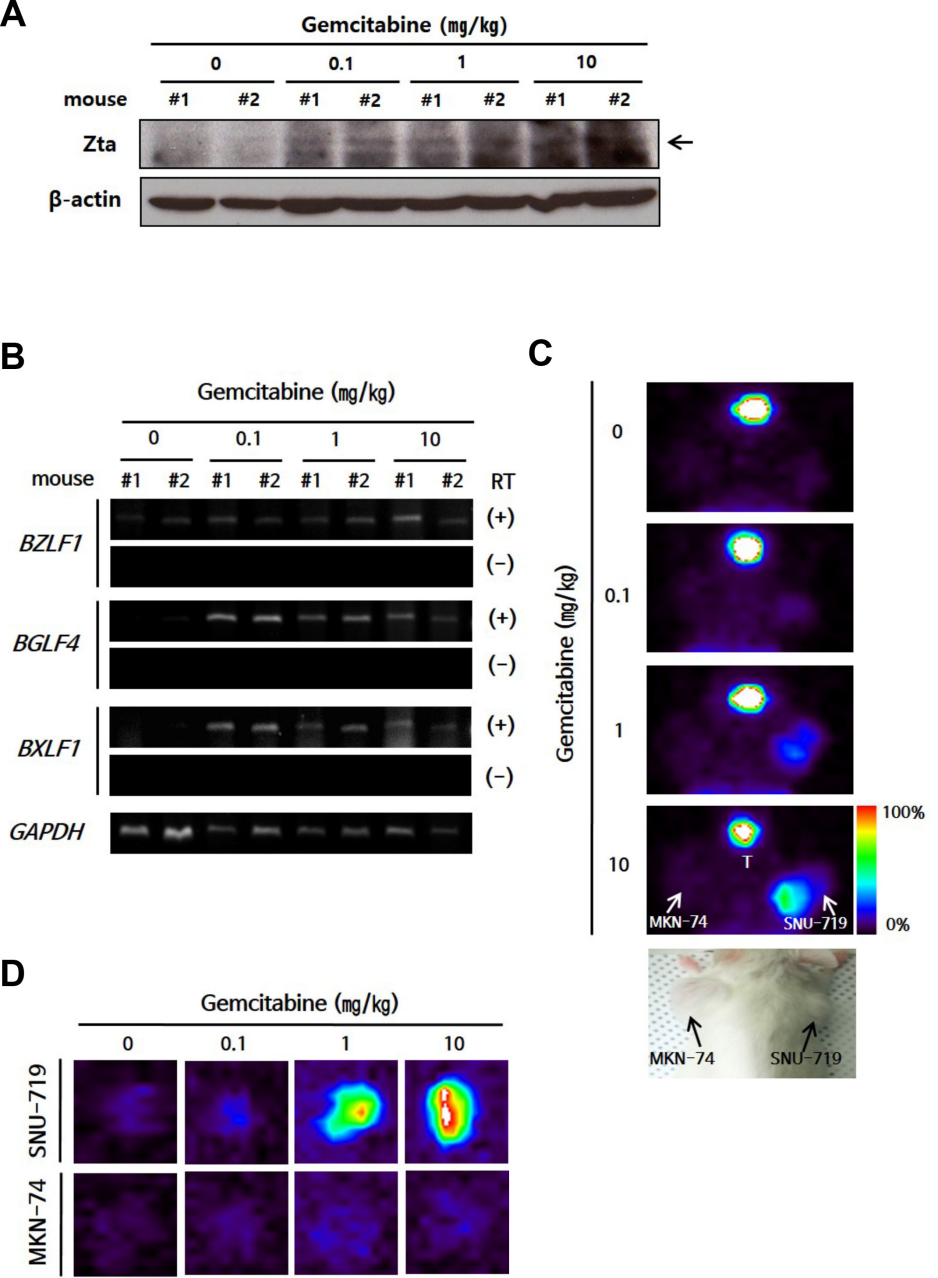
Establishment of a lytic activation-inducible EBVaGC mouse model **A.** Western blot and **B.** RT-PCR of EBV lytic genes in isolated tumors from gemcitabine-treated mice. *GAPDH* or β-actin was used as loading controls. **C–D.** Tumor cell-engrafted mice were injected with gemcitabine (0.1, 1, or 10 mg/kg) and administrated 200 μ Ci [^125^I] FIAU in 24 h after drug injection. The mice (C) or isolated tumors (D) were imaged using SPECT. Color bar indicates the range of [^125^I] FIAU uptake as a percentage. T, Thyroid.

Next, we tested whether gemcitabine induced functional EBV lytic proteins in this mouse model. The gemcitabine concentration commonly used in cancer therapy for human is 20–60 μM (*i.e*., 5.2–15.6 μg/ml) in plasma, and such levels are achieved by infusion of drug at a dose of 1,000–1,200 mg/m^2^ [29]. For our mouse model, gemcitabine was used with a much lower dose than used in cancer therapy, and induced Zta in SNU-719 cell-implanted tumors (referred to as SNU-719 tumors) in a dose-dependent manner (Figure 3A). *BGLF4* and *BXLF1* were also induced in SNU-719 tumors (Figure 3B) as observed in the *in vitro* system. We used [^125^I] FIAU-based single-photon emission computed tomography (SPECT) planar imaging to confirm the induction of functional EBV-TK/PK in SNU-719 tumors. The intensity of the [^125^I] FIAU signal showed a positive correlation with the dose of gemcitabine, whereas this signal was not detected in MKN-74 cell-implanted tumors (referred to as MKN-74 tumors) (Figure 3C). This result was confirmed by imaging of isolated tumors (Figure 3D).

### Efficient gemcitabine-GCV combination treatment in the EBVaGC mouse model

**Figure 4:**
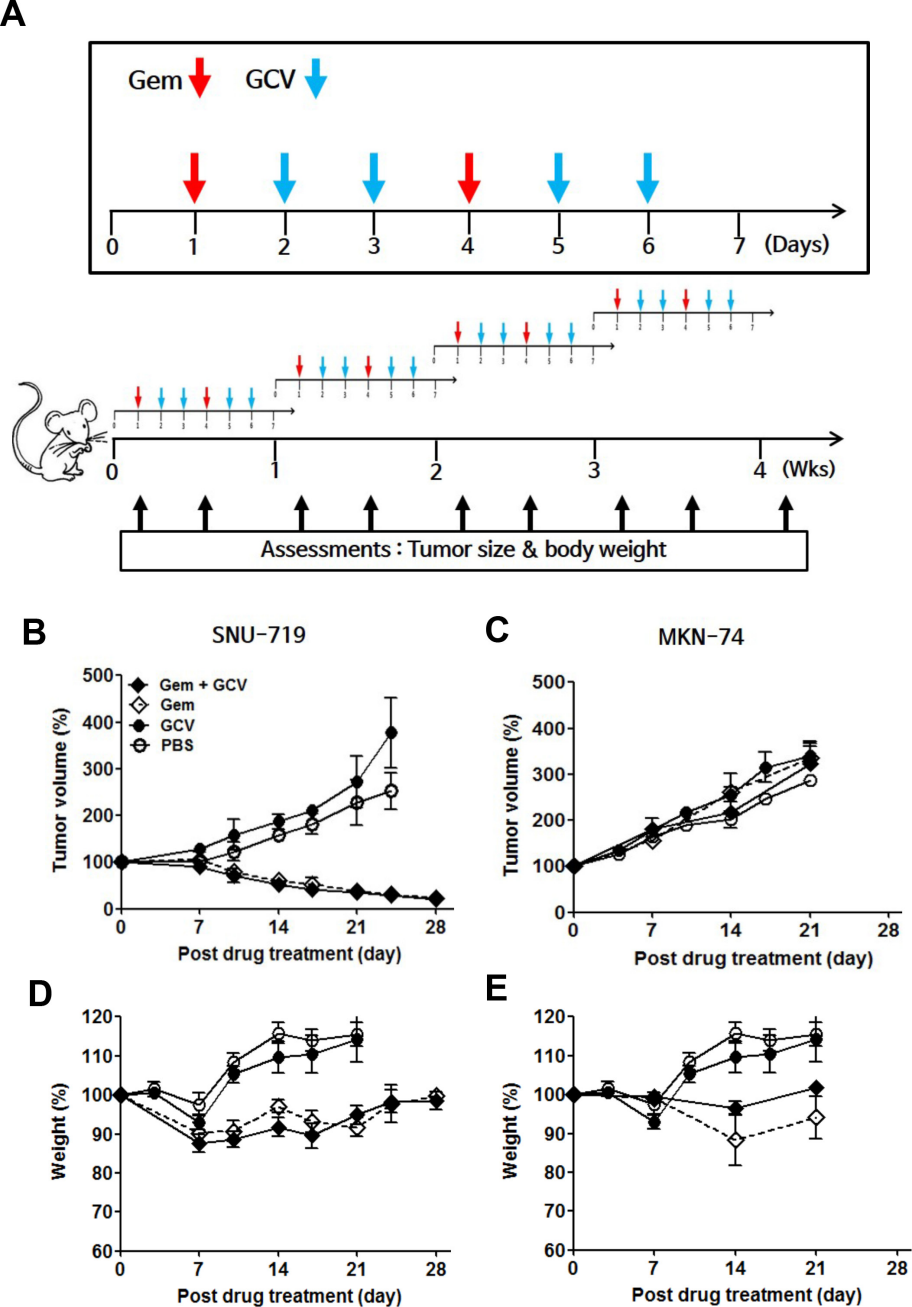
Gemcitabine-GCV combination treatment in EBVaGC cell-implanted mice **A.** Gemcitabine/GCV administration. Tumor volume in mice implanted with **B.** SNU-719 or **C.** MKN-74 cells and injected intraperitoneally with 10 mg/kg gemcitabine, 25 mg/kg GCV, or both according to the schedule shown in above. Body weight in **D.** SNU-719- or **E.** MKN-74-implanted mice injected intraperitoneally with 10 mg/kg gemcitabine, 25 mg/kg GCV, or both according to the schedule shown above. Tumor mass and body weight were measured before every gemcitabine injection. Each data point reflects observations from five mice. Both values were calculated as the percentage of the initial value for each mouse. Values represent means ± SEM.

**Figure 5:**
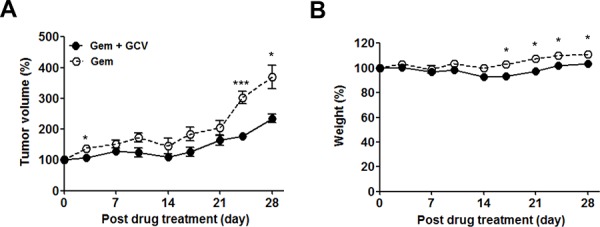
Optimization of gemcitabine-GCV combination treatment in EBVaGC mice Mice were injected with 0.5 mg/kg gemcitabine only or 25 mg/kg GCV in combination with 0.5 mg/kg gemcitabine as scheduled in Figure 4A. Tumor mass and body weight were measured before every gemcitabine injection. Each data point reflects observations obtained from five mice. Both values were calculated as the percentage of the initial value for each mouse. Values represent means ± SEM. *P < 0.05, **P < 0.01, ^***^P < 0.001

The ability of gemcitabine to induce EBV-TK/PK in the mouse model system led us to test combination treatment with GCV. Approximately 1 × 10^7^ SNU-719 cells or 2 × 10^6^ MKN-74 cells were engrafted subcutaneously in the right flank of NOD-SCID mice. When the tumors reached approximately 1,000 mm^3^, the mice were treated with the indicated dose of gemcitabine or GCV as scheduled (Figure 4A). A dose of 10 mg/kg gemcitabine and 25 mg/kg GCV had little influence on tumor growth in MKN-74 cell-engrafted mice; however, the same dose of gemcitabine, alone or in combination with GCV, resulted in gradual regression of tumors until the tumors were no longer palpable in SNU-719 cell-engrafted mice (Figure 4B and 4C). Mice treated with gemcitabine only and the gemcitabine-GCV combination lost body weight severely, which made the experiment discontinue after 3 cycles in accordance with IACUC guidelines (Figure 4D). Therefore, we sought to identify a gemcitabine concentration that produced synergism with GCV without inducing adverse effects. The combination of 0.5 mg/kg gemcitabine and 25 mg/kg GCV with the same schedule suppressed the growth of tumors significantly (Figure 5A), accompanied by significant but tolerable body weight loss (Figure 5B).

## DISCUSSION

The recent remarkable progress in cancer research has produced new target-oriented drugs and treatment strategies [32]. Three decades ago, virus-targeted therapies were tried for the treatment of virus-associated cancers [33]. An anti-viral prodrug, GCV, selectively phosphorylated by HSV-TK, was suggested for the treatment of virus-associated cancers [7]. EBV encodes TK and PK enzymes that each have the capacity to phosphorylate GCV and are only expressed during lytic activation. Therefore, chemicals that act as lytic inducers have been sought to facilitate treatment with GCV. Epigenetic modifying agents (5-azacytidine, trichostatin A, sodium butyrate, and valproic acid) and some anti-cancer drugs (5-fluorouracil [5-FU], cis-platinum, and taxol) induce lytic activation in EBV-positive cell lines that originate from various tumors [8, 9, 17, 19, 34, 35]. The effectiveness of these reagents differs among different cell types. For instance, 5-FU and cis-platinum efficiently induce lytic activation in EBV-positive epithelial cell tumors but not in lymphoblastoid cell lines (LCLs) or B cell tumors. These differences make it difficult to generalize the outcome of EBV-lytic induction treatments, and therefore only a few clinical trials of these agents have been performed [36, 37]. Recently, EBV lytic activation was reported to be induced by the ER or genotoxic stress response. Moreover, the ATM/p53 pathway that is activated during the genotoxic stress response directly influences Zta induction [18, 26]. The different sensitivity of lytic inducers among cell types may originate from differences in the underlying molecular mechanisms during lytic activation.

In this study, we attempted to find new drugs that induce lytic activation more efficiently in EBVaGCs and do not affect healthy cells and tissues by screening JHDL with *BZLF1* promoter-transfected AGS cells. This library consists of drugs that are already used in patients and approved for safety and toxicity, allowing a bypass of phase I/II clinical trials [38, 39]. Gemcitabine was selected and was confirmed as a lytic inducer by induction of lytic gene expression and EBV-TK/PK activity in EBVaGC SNU-719 cells. Moreover, we observed that an extremely low dose (5 ng/ml) of gemcitabine induced Zta in SNU-719 cells compared to the dose required in LCLs or B cell lines (1 μg/ml) [8], which was checked using our optimized schedule (Supplementary Figure S1C). The dose discrepancy between EBVaGC-derived cells and B cell lymphoma-derived cells requires further evaluation with respect to molecular mechanisms.

Furthermore, gemcitabine-induced lytic activation was evaluated to determine whether the ER or genotoxic stress pathway was involved. The ATM inhibitor KU55933, si-*ATM*, or si-*TP53* treatment induced a decrease in Zta protein expression. Since the duration of ATM inhibitor activity is very short, suppression by the ATM inhibitor was relatively weaker than that of si-*ATM*. Most Zta expression was diminished by si-*ATM* and si-*TP53*. Therefore, the ATM/p53 pathway may be a key regulator involved in lytic activation by gemcitabine. For this reason, p53 may be applicable as a biomarker to determine whether an EBVaGC patient is a candidate for gemcitabine-GCV combination treatment. *TP53* mutation is frequently observed in gastric cancers of various types, but is actually rarely observed in EBVaGCs [40, 41]. Moreover, the stability of p53 is also regulated by the interaction with EBNA1 [42]. Thus, the status of p53 may determine the responsiveness of gemcitabine-induced lytic activation, although this notion requires further evaluation.

The final goal of this study was to apply GCV to EBV-TK/PK-induced EBVaGC cells. The enzymatic activity of gemcitabine-induced EBV-TK/PK was verified by showing a positive correlation between gemcitabine concentration and the accumulation of [^125^I] FIAU. The accumulation of [^125^I] FIAU in the gemcitabine-untreated SNU-719 cells may be due to abortive lytic activation as discussed previously [8]. There are some data supporting abortive lytic activation in this study. Despite the absence of gemcitabine, *BZLF1* was observed by RT-PCR in SNU-719 cells and in mouse-implanted SNU-719 cells. Even with an extremely low dose of gemcitabine, the survival of SNU-719 cells was significantly decreased by GCV treatment, and this is consistent with a previous report [8]. Despite these observations, it remains unclear whether these observations are a direct read-out of abortive lytic activation.

We also evaluated the toxicity of gemcitabine and GCV respectively, and then examined the effects of an *in vitro* combination treatment. Gemcitabine exhibited a narrow safety window in SNU-719 and MKN-74 cells, but GCV was safe at a relatively high concentration (100 μg/ml). Synergistic effects of combination treatment were observed with a range of 0.1–100 ng/ml, as reported previously [8, 19]. The concentration of gemcitabine was the most important factor for efficacy of the combination treatment. In a previous report, the contribution of GCV-induced cytotoxicity may have been underestimated due to the high dose of gemcitabine (1 μg/ml) [8], as at this dose, we observed no beneficial outcomes in combination with GCV. To overcome this problem, we utilized multiple treatments of low-dose gemcitabine. Gemcitabine-GCV combination treatment has previously been reported as a treatment for cancers, albeit via a completely different mechanism [15]. Gemcitabine was utilized as a ribonucleotide reductase inhibitor, which reduces endogenous dGTP to increase the incorporation of phosphorylated GCV into DNA. Moreover, gemcitabine is also known to increase the sensitivity of bystander cytotoxicity, although the concentration of gemcitabine needed for such effects (10 μM or 2.6 μg/ml) is 100 times higher than the concentration used in our studies.

To date, only a few reports have described mouse models bearing EBVaGC-originated cell lines due to the rareness of EBV-naturally infected GC cell lines and the limited establishment of tumors in immune competent animals [19, 43] or nude mice [31]. In this study, we established a lytic activation-inducible EBVaGC mouse model using NOD-SCID mouse, and furthermore, [^125^I] FIAU-based EBV lytic activation monitoring system. Then, we evaluated a gemcitabine-GCV combination treatment with this EBVaGC mouse model. In the initial evaluation, gemcitabine was injected once a week, but the regression of tumor growth was slow in spite of the relatively high dose **(data not shown)**. Following the Ghosh *et al*. protocol for butyrate [44], we modified the dosing schedule to consider gemcitabine toxicity and rapid turnover rate, and observed the efficient induction of lytic activation with a multiple low dose schedule. As a result, we identified a combination treatment of 0.5 mg/kg gemcitabine and 25 mg/kg GCV that suppressed tumor growth more effectively than gemcitabine alone without adverse effects. To increase the efficacy of gemcitabine-GCV combination treatment, we would consider establishing new EBVaGC model using the nude mice due to the high sensitivity for drug-induced apoptosis of NOD-SCID mice. Furthermore, recently, it was reported that the combination treatment with gemcitabine, valproic acid, and GCV was efficient in EBV-positive refractory nasopharyngeal carcinoma patients [25]. Thus, additional combination with valproic acid could be considered.

In summary, gemcitabine was selected from a screen of JHDL for its ability to induce EBV lytic activation *in vitro* and *in vivo* in an EBVaGC cell line, SNU-719. ATM/p53 genotoxic stress is a key regulator of gemcitabine-induced lytic activation. We also developed a lytic activation-inducible EBVaGC mouse model to evaluate the efficacy of gemcitabine-GCV combination treatment *in vivo* in concert with an imaging system for evaluating lytic activation. EBV lytic activation-based GCV combination therapy showed promising results in our EBVaGC mouse model. To apply these results to EBVaGC patients, gemcitabine-induced lytic activation must be generalized using new patient-derived EBVaGC cell lines and their xenograft-bearing mouse models.

## MATERIALS AND METHODS

### Cell lines

The SNU-719 and MKN-74 cell lines, which were obtained from the Korea Cell Line Bank (Seoul, Korea), are EBV-positive and EBV-negative gastric carcinoma cell lines, respectively. Cells were cultured in RPMI-1640 supplemented with 10% heat-inactivated FBS (Hyclone, Tauranga, New Zealand) at 37°C in a humidified CO_2_ incubator.

### Chemical reagents and siRNAs

Gemcitabine (Eli Lilly, Indianapolis, IN, USA), ganciclovir (Sigma-Aldrich, St Louis, MO, USA), phorbol 12-myristate 13-acetate (PMA, 20 ng/ml; Sigma-Aldrich), and ataxia telangiectasia-mutated (ATM) kinase inhibitor (KU55933, 10 μM; Calbiochem, San Diego, CA, USA) were used. The siRNAs targeting *ATM* (NM_000051) and *TP53* (NM_000546) were designed and generated as Supplementary Table S2 (Integrated DNA Technologies; IDT, Coralville, IA, USA). The target sequences of the selected siRNAs are as follows: *ATM*, AGCUAUCAGAGAAGCUAAUAAAUTA and *TP53*, CCACCAUCCACUACAACUACA UGTG. The siRNAs were transfected using Lipofectamine RNAiMAX (Invitrogen, Carlsbad, CA, USA) according to the manufacturer's protocol.

### Induction and inhibition of EBV lytic activation in SNU-719 cells

Cells were treated with the indicated doses of gemcitabine (0–80 ng/ml) as shown in Figure 1A. Briefly, Cells were incubated for 24 h, then washed with PBS, followed by additional culture for 48 h. To suppress lytic activation, cells were treated with ATM inhibitor (KU55933) for 1 h after gemcitabine treatment.

### Western blot analysis

Cells or tumors were lysed with RIPA buffer (50 mM Tris-Cl [pH 7.5], 150 mM NaCl, 1% NP-40, 0.5% sodium deoxycholate, 0.1% SDS). Samples were separated on a 10% acrylamide gel and transferred to a 0.45 μm nitrocellulose membrane (Bio-Rad, Hercules, CA, USA). Membranes were blocked in TBS containing 5% non-fat milk and 0.05% Tween 20 solution and incubated with primary antibody (Ab). The following antibodies were used for western blot: anti-Zta (Argene, Verniolle, France), anti-p53 (Novocastra, Buffalo Grove, IL, USA), anti-p53 pSer15 (Cell Signaling Technology, Beverly, MA, USA), anti-ATM (Cell Signaling Technology), anti-ATM pSer1981 (Cell Signaling Technology), and anti-β-actin (Sigma-Aldrich)

### Immunofluorescence assay

Cells were fixed in cold 4% paraformaldehyde for 30 minute and blocked with 10% normal donkey serum (Jackson ImmunoResearch, West Grove, PA, USA) for 1 h at room temperature. Cells were stained with anti-Zta or anti-gp350 Ab (gift from Prof. Song) and Rhodamine Red-X-AffiniPure donkey anti-mouse IgG (H+L) (Jackson ImmunoResearch). and then visualized by LSM 700 confocal microscopy (Cal Zeiss, Oberkochen, Germany). In addition, 4, 6-diamidino-2-phenylindole (DAPI; Vector Laboratories, Burlingame, CA, USA) staining was also performed to visualize cell nuclei.

### RT-PCR

RNA was isolated from cell pellets or tumor tissues using the RNeasy Mini kit and RNase-free DNase (QIAGEN, Valencia, CA, USA) following the manufacturer's instructions. The cDNA was synthesized using 5 μg total RNA, SuperScript III reverse transcriptase (Invitrogen) and random hexamers. PCR was performed to evaluate EBV lytic gene expression using the specific primers: BZLF1, 5′-ACC AAG CCG GGG GAG AAG CA-3′ and 5′-CCA GGC TTG GGC ACA TCT GC-3′; BGLF4, 5′-CGC TCG GCT ACT CGC TGC TC-3′ and 5′-CGG AGG AAG CGG GCA AAC GT-3′; BXLF1, 5′-TTA CCC TGC CCA GGG GAG CC-3′ and 5′-GTC ATC GAG CCC AAG GCC GG-3′; GAPDH, 5′-GAT GGC ATG GAC TGT GGT CA-3′ and 5′-GCA ATG CCT CCT GCA CCA CC-3′. GAPDH expression was used as an internal control. RT (−) PCR was performed to rule out contamination by EBV genomic DNA.

### [^125^I] FIAU cellular uptake assay

Cells, which were induced into lytic activation as described, were incubated with 1 μCi/2 ml [^125^I] FIAU at 37°C for 4 h. Then cells were washed and harvested with 200 μl trypsin-EDTA (0.25%, Thermo Fisher Scientific Inc., Rockford, IL, USA), followed by addition of 400 μl PBS. The radioactivity was measured using a γ-counter (Perkin Elmer, Waltham, MA, USA). The accumulation of [^125^I] FIAU was calculated as the percentage of the input dose added to the medium (%AD) [45].

### Cell viability assay

Cell viability was analyzed using the Cell Counting Kit-8 (CCK-8; Dojindo Lab, Kumamoto, Japan). SNU-719 (3 × 10^3^ cells/100 μl) and MKN-74 (2 × 10^3^ cells/100 μl) cells were plated in 96-well plates and incubated at 37°C in 5% CO_2_ overnight. Then, the cells were treated as described in Figure 2D. Then, 10 μl CCK-8 solution (2-[2-methoxy-4-nitrophenyl]-3-[4-nitrophenyl]-5-[2, 4-disulfophenyl]-2H-tetrazolium) was added to each well. The plates were incubated at 37°C for 3 h, and the absorbance at 450 nm was measured using a microplate reader (Perkin Elmer, Waltham, MA, USA).

### Generating EBV-positive or -negative GC cell line-implanted mice

All mice were maintained under specific pathogen-free conditions, and the experiments involving animals were approved by the Institutional Animal Care and Use Committees (IACUC) at Yonsei University College of Medicine (2012-0263). Six to seven-week-old female NOD-SCID mice (Korea Research Institute of Bioscience and Biotechnology, Daejeon, Korea) were used for all experiments. SNU-719 or MKN-74 cells were suspended in a 200 μl solution containing 100 μl Matrigel (BD Biosciences, Franklin Lakes, NJ, USA) and 100 μl PBS and then injected subcutaneously into the right or left flank of mice. Tumor size was measured approximately every third day with calipers, and tumor volume was calculated as *l* × *w^2^* (*l*: long axis, *w*: width) [15]. Approximately 6 or 4 weeks after implantation of SNU-719 or MKN-74 cells, respectively, mice carrying tumors that reached a volume of approximately 1,000 mm^3^, were used for *in vivo* experiments and imaging.

### Molecular imaging of *in vivo* lytic activation

When the tumor size reached approximately 1,000 mm^3^, 200 μCi [^125^I] FIAU was administered to each tumor-carrying mouse through the tail vein. The mice were imaged by [^125^I] FIAU-based SPECT planar imaging as previously described [45]. Briefly, the mice were placed in a posterior position on a warm-bed and anesthetized with 2% isoflurane (Choongwae, Seoul, Korea) before injection of [^125^I] FIAU and 1.5% isoflurane during imaging. For thyroid-blocked images, 1 mg sodium perchlorate (Sigma-Aldrich) was injected intraperitoneally into the mice before injection of [^125^I] FIAU.

### Statistical analysis

Statistical analysis was performed with unpaired Student's *t*-tests. All *in vitro* experiments were performed for at least three times. Values represent means ± SEM. *P < 0.05, **P < 0.01, ^***^P < 0.001.

## SUPPLEMENTARY MATERIALS AND METHODS




