# Supplementary material for: Targeted therapy for Epstein-Barr virus-associated gastric carcinoma using low-dose gemcitabine-induced lytic activation
Source: Oncotarget. 2015 Sep 4;6(31):31018–29. doi: 10.18632/oncotarget.5041 (PMC4741585; doi:10.18632/oncotarget.5041)
Supplement: Supplementary file 1 [file oncotarget-06-31018-s001.pdf]

SUPPLEMENTARY MATERIALS AND METHODS

Cell lines

LCL-1 is an immortalized EBV-positive lymphoblastoid cell line [46]. Cells were cultured in RPMI-1640 supplemented with 10% heat-inactivated FBS (Hyclone) at 37°C in a humidified CO_2 incubator.

Western blot analysis

The following antibodies were used for western blot: anti-GRP78 (Santa Cruz) and anti-CHOP (Santa Cruz).

Flow cytometry

Mice were sacrificed. Then tumors were isolated and minced to single cell suspension. Cells were treated with RBC lysis buffer, followed by washing with PBS. Cells were stained with FITC-labeled anti-H-2Kd Ab (BD Biosciences, San Diego, CA, USA) and APC-labeled anti-HLA-ABC Ab (BD Biosciences) for 30 min at 37°C, followed by washing with PBS. Stained cells were analyzed with FACS LSR II flow cytometer (BD Biosciences), and the FACS data were analyzed by FACSDiva software (BD Biosciences).

EBER-ISH

Sections (4-µm thick) from formalin-fixed and paraffin-embedded blocks were prepared. EBER-ISH was performed by Ventana ISH iView kit (Ventana, Tucson, AZ, USA). Paraffin-embedded tissue sections were deparaffinized using EZ Prep buffer (Ventana), followed by digestion with Protease I for 4 min. The tissues were denatured at 85°C (10 min) and hybridized with fluorescein-labeled EBER-specific probes at 37°C (1 h). After hybridization, tissues were washed for 15 min at 57°C (3 times, 2x saline sodium citrate buffer) and incubated with anti-fluorescein mAb for 20 min. Finally, the Alkaline Blue Detection kit (Ventana) was used according to the manufacturer's protocol. The slides were counterstained with Nuclear Fast Red for 10 min.

SUPPLEMENTARY FIGURES AND TABLES

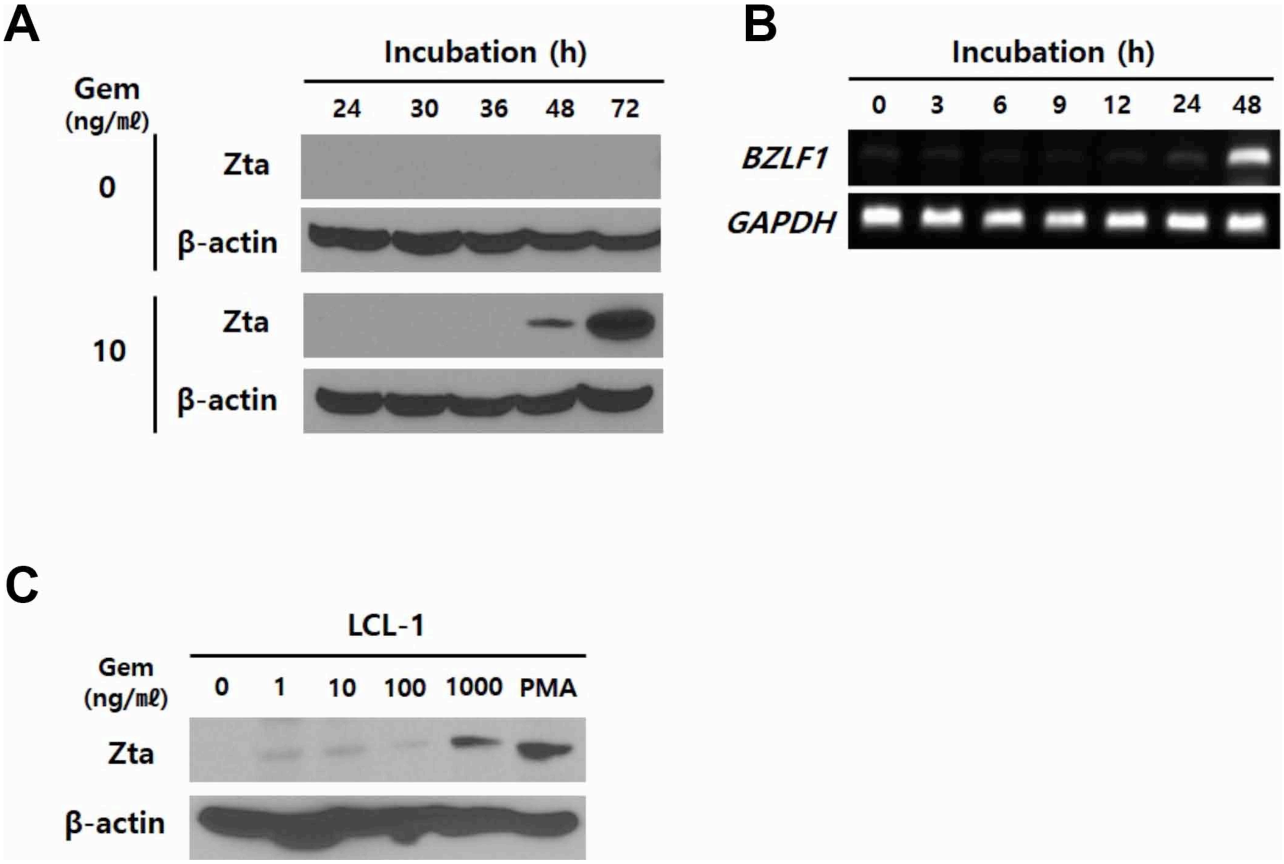

Supplementary Figure S1: Gemcitabine-induced EBV lytic activation. SNU-719 cells were treated with 10 ng/ml gemcitabine, and then harvested after the indicated incubation times. Western blot **A.** or RT-PCR **B.** was performed to visualize Zta expression. **C.** LCL-1 cells were treated with gemcitabine (0–1 μg/ml). Zta expression was evaluated by western blot. β-actin was used as a loading control.

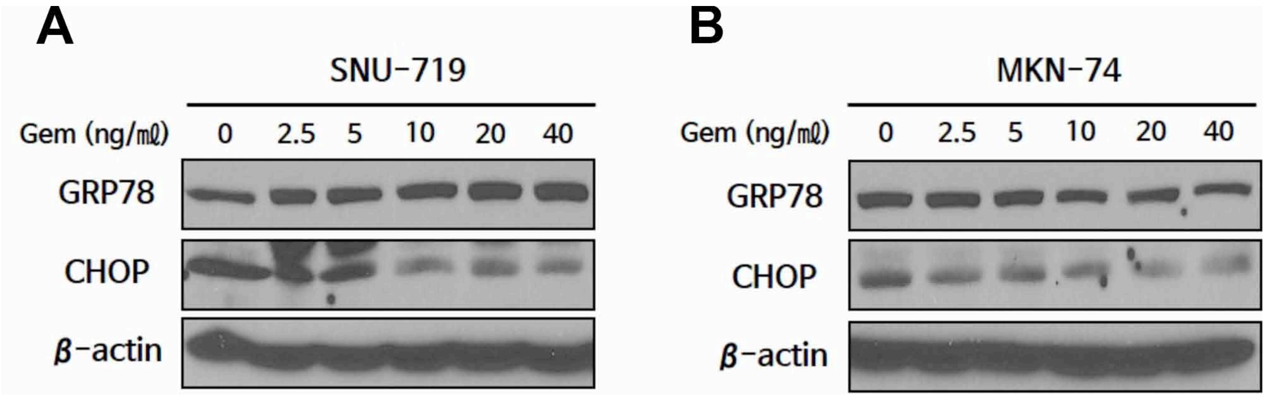

Supplementary Figure S2: ER stress response in gemcitabine-induced lytic activation. SNU-719 **A.** and MKN-74 **B.** cells were treated with gemcitabine (0–40 ng/ml). Western blot was performed to visualize GRP78 and CHOP expression. β-actin was used as a loading control.

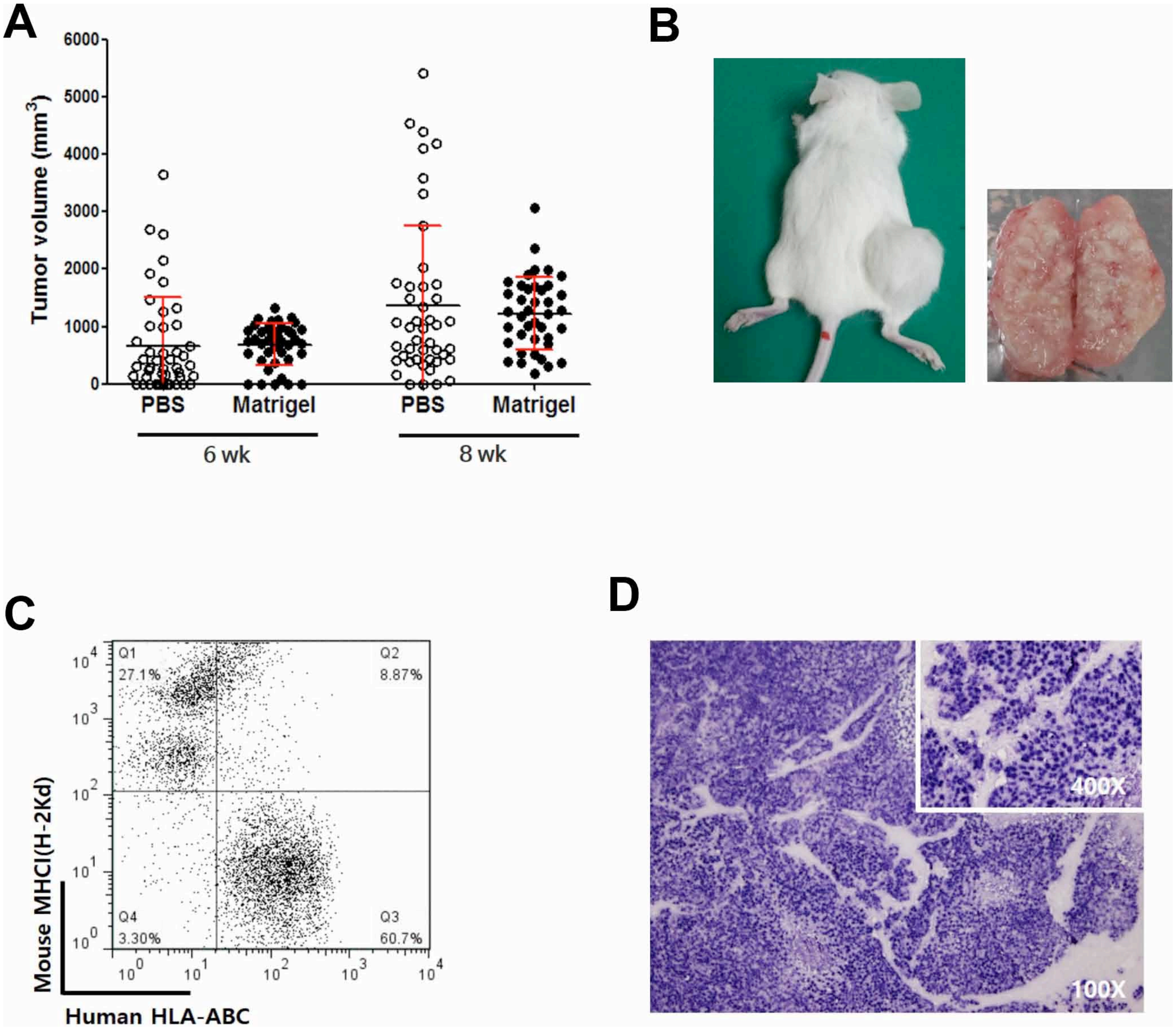

Supplementary Figure S3: Establishment of SNU-719 tumors in NOD-SCID mice. A. SNU-719 cells suspended in Matrigel or PBS were injected subcutaneously into the right or left flanks of mice. Tumor mass was measured 6 or 8 weeks after implantation. The mean (black bar) and standard deviation (red bar) are indicated. **B.** Representative implanted tumor in the flank of NOD-SCID mouse (left) and the cross-sectional view of isolated tumor (right). Flow cytometry **C.** and EBER-ISH **D.** of isolated tumor.

Supplementary Table S1: List of drug candidates selected from high-throughput screening

Supplementary Table S2: siRNA sequences used to targets

Target	siRNA no.	siRNA sequence
ATM	**1**	**AGCUAUCAGAGAAGCUAAUAAAUTA**
	2	ACAAGUGUAAUAUGGACAGUAUCTA
	3	GCUGCGGAGAUUAACAAAUGGGUGA
TP53	**1**	**CCACCAUCCACUACAACUACAUGTG**
	2	GGAUUUCAUCUCUUGUAUAUGAUGA
	3	GGAUGUUUGGGAGAUGUAAGAAAATG
